# Analysis of Medical Students’ Motivation: Insights into the Development of Future Health Professionals

**DOI:** 10.3390/bs16010097

**Published:** 2026-01-12

**Authors:** Karina Iveth Orozco-Jiménez, María Alejandra Samudio-Cruz, Jonatan Baños-Chaparro, Eleonora Ocampo-Coronado, Ileana Chávez-Maisterra, Marcela María José Rodríguez-Baeza, Benjamín Gómez-Díaz, María Valentina Toral-Murillo, Elvira Rodríguez-Flores, Melissa Fernández-Torres, Ana Cecilia Corona-Pantoja, Mariana Selene de Alba-Torres, Luz Berenice López-Hernández

**Affiliations:** 1 Unidad Académica de Medicina, Universidad Autónoma de Guadalajara, Av Patria 1201, Zapopan 45129, Mexico; iveth.orozco@edu.uag.mx (K.I.O.-J.); eleonora.ocampo@edu.uag.mx (E.O.-C.); ileana.chavez@edu.uag.mx (I.C.-M.); ana.ceciliacorona@edu.uag.mx (A.C.C.-P.); mariana.alba@edu.uag.mx (M.S.d.A.-T.); 2 Instituto Nacional de Rehabilitación Luis Guillermo Ibarra Ibarra, Mexico City 14389, Mexico; psic.alejandra.samudio@gmail.com (M.A.S.-C.); bgodiaz@gmail.com (B.G.-D.); 3 Programa Académico de Psicología, Facultad de Ciencias de la Salud, Universidad Privada Norbert Wiener, Lima 15046, Peru; jonatan.banos@uwiener.edu.pe; 4 Unidad Académica de Ciencias de la Salud, Universidad Autónoma de Guadalajara, Av. Patria 1201, Zapopan 45129, Mexico; marcela.rbaeza@academicos.udg.mx (M.M.J.R.-B.); valentina.toral@edu.uag.mx (M.V.T.-M.); elvira.rodriguez@edu.uag.mx (E.R.-F.); melissa.fernandez@edu.uag.mx (M.F.-T.); 5 Departamento de Biología Celular y Molecular, Centro Universitario de Ciencias Biológicas y Agropecuarias (CUCBA), Universidad de Guadalajara, Zapopan 44100, Mexico

**Keywords:** motivation, engagement, medical students, stress

## Abstract

Medical students experience fluctuations in their motivation, influenced by various factors, including curricular rigor, mental health, and institutional factors. Based on Self-Determination Theory (SDT) and the Four Pillars of Academic Engagement (HPEE), this study, conducted at a private Mexican university, examined motivational variation according to academic year, curricular impact, gender differences, and its relationship with mental health. Methods: A quantitative, cross-sectional, descriptive study was conducted using qualitative tools for contextualization (n = 1326). Mann–Whitney U tests, Kruskal–Wallis tests, logistic regression, and psychological network analysis were performed. Results: Motivation showed cross-sectional variation: high in preclinical years 1 and 2, decreasing in clinical years 3 and 4 (*p* < 0.001), and rebounding in year 6. The reformed curriculum (elective subjects, student-centered active learning) resulted in greater motivation (OR = 10.68, *p* < 0.001). Women tended to have slightly higher motivation (*p* = 0.050), higher grade point averages (*p* < 0.001), but also greater stress (*p* < 0.001). Network analysis revealed that intrinsic achievement (centrality = 1.11) and curiosity about knowledge (predictability = 84.5%) are the main drivers, while demotivation was linked to the later years. The qualitative part of the study showed altruism/curiosity as the main motivators; mistreatment/workload (demotivators). Conclusions: Motivation is context-sensitive, peaks in the preclinical stage, and recovers with autonomy but is vulnerable during clinical immersion. Autonomy in course selection, active student-centered pedagogies, and gender-sensitive support foster sustained participation. The centrality of intrinsic factors in the network highlights that achievement motivation and knowledge are general and independent motivators. Qualitative data reveal systemic barriers. Stage-specific interventions, such as mentoring, student support programs, and reporting mistreatment, can be crucial for strengthening resilience and performance. Longitudinal and multi-institutional studies are needed to validate the causality and generalizability of this study.

## 1. Introduction

Higher education institutions (HEIs) prepare future medical doctors to meet the complex demands of modern healthcare, requiring not only medical knowledge and clinical skills but also motivation, resilience, and ethical competence (Bin Abdulrahman et al., 2023). Medical education presents unique challenges, including rigorous curricula, intensive clinical rotations, and emotional stressors that challenge students’ motivation, engagement, and performance (Bergmann et al., 2019). Understanding the psychological and contextual factors influencing these outcomes is critical for developing resilient, competent professionals. This study applies Self-Determination Theory (SDT; Deci & Ryan, 2000) which posits autonomy, competence, and relatedness as core drivers of intrinsic motivation and the Four Pillars of Academic Engagement (FPAE; Bowden et al., 2021) to explore motivational dynamics across medical training.

### 1.1. Self-Determination Theory

**Figure 1:**
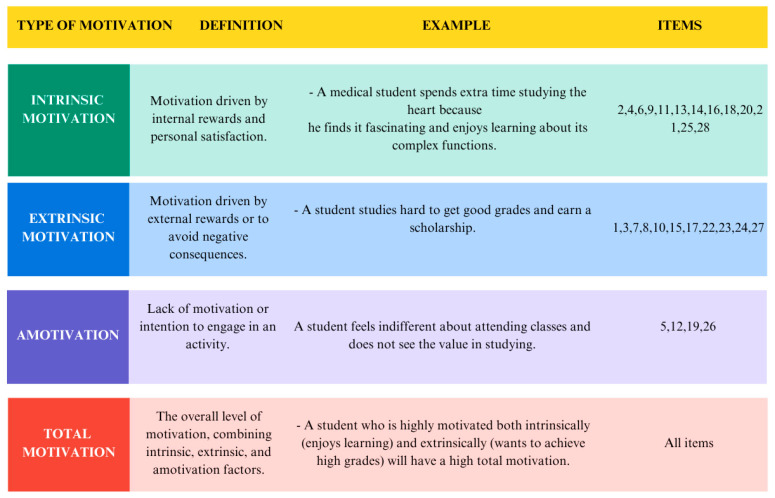
Details AMS subscales and items.

Motivation is the central process that initiates, guides, and sustains goal-oriented behaviors, acting as the inner force that drives people to satisfy their needs and goals. The SDT offers a robust framework for understanding motivation in medical education by emphasizing the fulfillment of three psychological needs: autonomy (self-directed choice), competence (perceived capability), and relatedness (belonging and connection with others). The STD also distinguishes two main forces: intrinsic motivation, which arises from genuine interest in and inherent enjoyment of an activity for its own sake, and extrinsic motivation, which stems from external factors such as the pursuit of rewards or the avoidance of punishment or pressure. At the opposite end of the motivational continuum lies amotivation, characterized by the absence of any drive or value toward the activity, implying a lack of belief in one’s own competence or in achieving a desired outcome (Figure 1). SDT further emphasizes that autonomous motivation (which includes intrinsic and more internalized forms of extrinsic motivation) directly strengthens self-efficacy—that is, the student’s belief in their own ability to perform the behaviors necessary to achieve their goals. High self-efficacy predicts greater persistence, effort, and deeper engagement with complex tasks. Therefore, the satisfaction of psychological needs acts as a key mechanism that interrelates motivation, engagement, and ultimately, academic performance (SDT; Deci & Ryan, 2000).

### 1.2. Four Pillars of Academic Engagement

The Four Pillars of Academic Engagement (FPAE) framework, presented by Bowden et al. (2021), conceptualizes student engagement as a multidimensional construct encompassing behavioral, affective, cognitive, and social dimensions. This model empowers educators to design learning environments that promote deep and sustained participation, going beyond the mere passive transmission of content (Bowden et al., 2021). This is crucial for maintaining student motivation. Each dimension interacts dynamically to influence student success:

Behavioral Engagement: Reflects observable participation and adherence to academic norms. Including class attendance, it also encompasses proactive actions such as completing preparatory readings, submitting assignments on time, and contributing to discussions. For example, a student who asks for clarification during office hours or attends peer tutoring sessions exemplifies strong behavioral commitment, indicating active participation in the learning community. Although this pillar primarily corresponds to extrinsic motivation (specifically external regulation), regular class attendance can foster a sense of belonging and social connection, making motivation more lasting.

Affective Commitment: This encompasses emotional investment in the institution, classmates, professors, and the course. It manifests as a sense of belonging, enthusiasm, and pride in achievements. Instructors foster this pillar through inclusive environments that normalize mistakes as learning opportunities and by linking the material to professional goals; for example, by illustrating how biochemistry underpins clinical diagnosis. This pillar largely corresponds to autonomous (identified intrinsic) motivation.

Cognitive Commitment: This involves a psychological investment in mastering complex concepts. It prioritizes critical thinking, synthesis, and self-regulation over rote memorization. A cognitively engaged student explores underlying mechanisms (e.g., physiological principles) and applies them to novel scenarios, such as multidisciplinary case analyses. This pillar is predominantly linked to autonomous motivation or genuine curiosity for learning.

Social engagement: This emphasizes the collaborative construction of knowledge through meaningful interactions. Team-based problem-based learning (PBL), where students integrate diverse perspectives to solve real clinical cases, improves both academic outcomes and interpersonal competence (Bowden et al., 2021; Chavez-Maisterra et al., 2024). Together, these pillars provide a diagnostic and interventional perspective for optimizing engagement in medical education, with differential impacts on retention, performance, and well-being. This pillar is predominantly linked to intrinsic motivation.

For example, problem-based learning (PBL) interventions enhance autonomy and team cohesion by assigning clear roles, enforcing systematic participation, and addressing communication barriers, even in resource-constrained settings (Atreya et al., 2025). Such structured autonomy optimizes active involvement and resource use among learners with heterogeneous preparation levels. Competence is bolstered through specific, process-oriented feedback (Bouffard, 2017). Low-fidelity simulations such as venipuncture practice further build procedural confidence before high-stakes clinical encounters.

Relatedness, balance the isolation inherent in medical training. Regular faculty check-ins on student well-being foster belonging, while flexible, interdisciplinary PBL models integrating real-world scenarios strengthen collaborative engagement (Mebert et al., 2020). Empirical support confirms these mechanisms: autonomous motivation significantly predicts academic engagement and performance, with commitment to learning mediating this pathway (Wu et al., 2020). Thus, SDT and FPAE provide a comprehensive perspective for understanding how need satisfaction drives adaptive motivational processes in medical education.

### 1.3. Interrelationship Between Self-Determination Theory, Academic Engagement, and Motivation in Medical Education

According to SDT, satisfaction of the basic psychological needs for autonomy, competence, and relatedness fosters intrinsic and autonomous motivation, which in turn activates the four pillars of academic engagement: behavioral, emotional, cognitive, and social (Bowden et al., 2021). In the highly demanding context of medical education, this multidimensional engagement serves as a critical mediator between autonomous motivation and academic performance (Orsini et al., 2018; Wu et al., 2020). Specifically, autonomy and competence satisfaction promote greater active participation, sustained effort, persistence, and the use of deep learning strategies, whereas relatedness satisfaction reduces isolation and enhances enjoyment, enthusiasm, and sense of belonging.

These positive effects are amplified by key mediators such as academic self-efficacy (Artino, 2012), resilience to academic stress (Neufeld & Malin, 2019; Zawiślak et al., 2024), and psychological well-being (Mansfield et al., 2020), which protect against burnout and sustain engagement in the face of chronic stressors (González & Paoloni, 2014; Ten Cate et al., 2011).

Contextual and pedagogical factors—such as problem-based learning (PBL) with autonomous case selection, structured process-focused feedback (e.g., on procedural skills such as suturing), clinical simulations, and interpersonal support—strengthen the satisfaction of these three basic psychological needs and optimize engagement even in resource-constrained settings (Atreya et al., 2025; Bouffard, 2017; Mebert et al., 2020).

Consistent empirical evidence, including a large-scale study of 1930 Chinese medical students, demonstrated that intrinsic and autonomous motivation predicts higher academic engagement and better performance than controlled motivation or amotivation, with engagement exerting significant mediation (Wu et al., 2020). Students with high intrinsic motivation and low controlled motivation—driven by genuine interest rather than external pressures such as family expectations or status—adopt deeper learning strategies, devote more time to self-study, achieve higher grades, and experience less burnout (Kusurkar et al., 2013; Orsini et al., 2018). Conversely, predominantly controlled motivation or amotivation is associated with superficial learning and poorer academic outcomes.

Resilience defined as the capacity to adapt to stressors such as rigorous clinical assessments and workload overload sustains motivation and engagement by buffering burnout (González & Paoloni, 2014; Ten Cate et al., 2011). Competence satisfaction is directly linked to greater resilience among medical students (Neufeld & Malin, 2019), and autonomous motivation is associated with better quality of life and higher resilience (Zawiślak et al., 2024). Similarly, academic self-efficacy—the belief in one’s ability to accomplish required tasks—enhances motivational and engagement processes (Artino, 2012). Pedagogical interventions that foster autonomy (e.g., PBL) and provide assertive, competence-focused feedback increase perceived self-efficacy, yielding more motivated, engaged, and resilient students. However, although autonomous motivation predicts self-efficacy, its direct effect on academic performance becomes non-significant when engagement mediation is accounted for (Wu et al., 2020).

In conclusion, integrating SDT with the four-pillar model of academic engagement provides a robust theoretical framework that elucidates dynamic, bidirectional interactions among autonomous motivation, multidimensional engagement, self-efficacy, and resilience. This framework offers a solid foundation for designing pedagogical interventions in medical training that prevent demotivation, simultaneously promote deep learning and well-being, and indirectly optimize academic performance through the complex interplay of these constructs within the demanding curricular context.

### 1.4. Academic Performance as an Outcome of Motivation and Engagement

Academic performance in medical education emerges from the interplay of prior achievement, self-regulated learning (SRL), psychological well-being, and sustained intrinsic motivation underpinned by deep learning strategies. Prior academic achievement—indexed by entrance examinations and secondary school grades—serves as the strongest baseline predictor of success (Gebru & Verstegen, 2023).

Beyond foundational ability, self-regulated learning (SRL) the proactive planning, monitoring, and adaptation of study behaviors significantly enhances outcomes. For instance, students who construct concept maps integrating microbiology and pathology demonstrate superior knowledge synthesis and performance (Cho et al., 2017). This underscores that how students learn is as critical as what they learn.

Psychological well-being, including stress management and self-efficacy, further mediates success. Students with robust mental health and confidence better navigate the cognitive and emotional demands of medical training. Conversely, depression, anxiety, and burnout impair performance, though moderate anxiety may occasionally heighten focus (Mansfield et al., 2020). Intrinsic motivation, driven by curiosity and personal value, sustains effort and fosters deep learning approaches—prioritizing understanding over memorization—which buffer motivational decline across training years (Mansfield et al., 2020). Social and environmental factors, including peer support, teaching quality, and institutional climate, amplify these effects.

The objective of the present study was to gain insights into motivation across training stages and evaluate the influence of curricular innovations, gender differences, and mental health on academic engagement using Self-Determination Theory (SDT) and the Four Pillars of Academic Engagement (FPAE) as guiding frameworks.

## 2. Materials and Methods

### 2.1. Setting

This cross-sectional study was conducted at the Autonomous University of Guadalajara (UAG), a private Catholic institution in Mexico with campuses in Jalisco (enrollment: 10,124 students) and Tabasco (enrollment: 1895 students). UAG’s medical program, its most popular offering, comprises two tracks: the National (“Latino”) Program (taught in Spanish, primarily for Mexican and Latin American students) and the International Program (taught in English, for global students). The study focused exclusively on the Latino Program at the Guadalajara campus, including students from all years except the fifth, who were off campus for mandatory hospital internships.

In 2023, UAG implemented a revised curriculum incorporating elective courses and active learning methods, such as simulation, problem-based learning (PBL), gamification, and technology-enhanced tools. At the time of data collection (January–December 2024), first- and second-year students experienced this new curriculum, while third- through sixth-year students followed the original program.

### 2.2. Participants and Procedures

The study protocol was approved by the UAG University Research Coordination. Data were collected from January to December 2024. Participants included 1326 medical students enrolled in the UAG Latino Program at Guadalajara, recruited via non-probability convenience sampling. Recruitment occurred through in-person sessions, where faculty explained the study objectives, addressed queries, and invited voluntary, anonymous participation via an online questionnaire. Exclusion criteria included duplicate submissions, incomplete responses, and non-participation (e.g., absences on administration days or drop-outs). Fifth-year students were excluded due to off-campus internships, limiting on-site explanation and support. Of the 1326 participants, 212 were further excluded from network analysis due to missing data, yielding a final analytic sample of 1114 for that component.

### 2.3. Instrument

Academic motivation was assessed using a 28-item Spanish version of the Academic Motivation Scale (AMS), grounded in Self-Determination Theory (Deci & Ryan, 2000). This version was reportedly adapted and validated for medical students (Al Ansari et al., 2021) and was also available in Spanish. The original 7-point Likert scale was shortened to 5 points (1 = strongly disagree to 5 = strongly agree) following pilot testing, which suggested improved response reliability. Reliability was evaluated in a pilot sample of 56 UAG medical students from the same program and campus, yielding a Cronbach’s α of 0.89 overall. McDonald’s ω estimates for subscales were as follows: intrinsic motivation to know (0.92), toward accomplishment (0.81), to experience stimulation (0.84), identified regulation (0.88), introjected regulation (0.86), external regulation (0.82), and amotivation (0.84)—all acceptable to excellent. Perceived stress was self-reported on a 10-point scale (1 = no stress to 10 = extreme stress), without physiological corroboration (e.g., cortisol assays). Mental health status was assessed via a binary self-report (“yes/no” for current professionally diagnosed condition), without details on diagnosis, severity, or history. Academic performance was self-reported as overall grade average (0–10 scale). Professional interest was rated on a 10-point scale (1 = no interest to 10 = extreme interest).

### 2.4. Data Processing and Statistical Software

Analyses were conducted using Jamovi (version 1.6.23; (Şahin & Aybek, 2020)), IBM SPSS Statistics (version 26.0; IBM Corp., 2019), JASP (version 0.14.1; Love et al., 2019), and RStudio (version 1.4.1717; R Core Team, 2021) with relevant packages (e.g., bootnet, networktools, qgraph, mgm). Significance was set at α = 0.05.

#### 2.4.1. Exploratory Data Analysis

Quantitative variables were summarized with means, standard deviations, and distributions assessed via Kolmogorov–Smirnov and Shapiro–Wilk tests, histograms, and Q-Q plots. Most data were non-normal, guiding nonparametric test selection. Categorical variables were reported as frequencies and percentages.

#### 2.4.2. Inferential Statistical Tests

**Figure 2:**
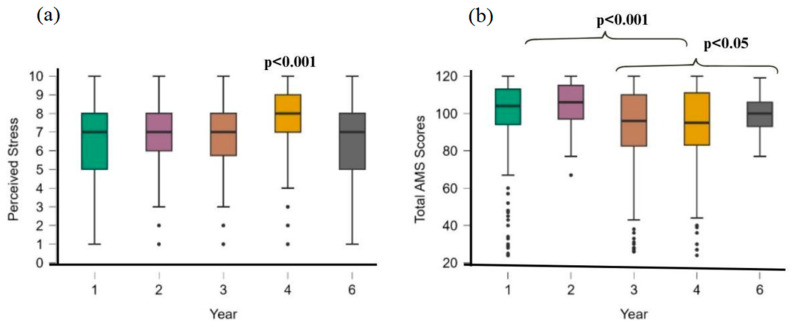
Kruskal–Wallis tests of years of study, perceived stress, and AMS scores. (**a**) Comparison of perceived stress and the year of study, (**b**) comparison of total AMS scores and years of study.

Group comparisons used Mann–Whitney U (two groups, e.g., stress by mental health diagnosis) and Kruskal–Wallis H (Figure 2), with Bonferroni-corrected post hoc tests for significant effects associations between categories (e.g., gender and mental health) employed χ^2^ tests, with odds ratios (ORs) and 95% confidence intervals (CIs) for significant findings.

#### 2.4.3. Regression Analysis

Multiple linear regression predicted continuous outcomes (e.g., motivation, performance), and binary logistic regression modeled dichotomous ones. Assumptions (linearity, homoscedasticity, multicollinearity) were verified; results included adjusted ORs (aORs) with 95% Cis (Table 1).

### 2.5. Psychological Network Analysis

Networks were estimated following cross-sectional guidelines (Burger et al., 2023). Sociodemographics used frequencies/percentages; psychological variables used means/standard deviations. Redundant nodes (r > 0.50) were detected via Goldbricker (networktools; (Hittner et al., 2003; Jones, 2018)). Networks employed ggmModSelect with Spearman correlations (bootnet; Isvoranu et al., 2022), selecting optimal Gaussian graphical models via EBIC (100 iterations). Visualization used Fruchterman-Reingold layout (qgraph; (Epskamp et al., 2012)), with edge thickness denoting strength. Local metrics included expected influence (EI; qgraph) and predictability (R^2^; mgm; (Haslbeck & Waldorp, 2020)). Global topology assessed density, transitivity, and small-world index (Isvoranu et al., 2022). Stability used nonparametric bootstraps (1000 iterations) for edge CIs and case-dropping (CS > 0.25; Burger et al., 2023). Group comparisons (e.g., by sex/diagnosis) applied Pearson correlations and Network Comparison Test (NCT).

### 2.6. Open-Ended Questions Analysis

Responses to two open-ended questions—“What motivates you to learn in the medical field?” and “What demotivates you?”—were thematically analyzed using ATLAS.ti software (Version 24.1.0). Coding was guided by SDT (Deci & Ryan, 2000) and the Four Pillars of Academic Engagement (FPAE; Bowden et al., 2021).

## 3. Results

### 3.1. Participants and Descriptive Statistics

The study included 1326 medical students from the UAG Latino Program (863 women, 463 men). Most participants were aged 18–21 years (n = 918), with the remainder aged 22 years or older (n = 408). A notable proportion reported a diagnosed mental health condition (n = 506), while the majority did not (n = 820). Descriptive statistics for key variables revealed moderate stress levels (M = 6.72, SD = 2.01), strong academic performance (M = 8.70, SD = 0.60), and moderate overall motivation as measured by the Academic Motivation Scale (AMS; M = 99.76, SD = 17.66).

### 3.2. Motivation

Cross-sectional analyses indicated a nonlinear pattern in motivation across academic years: high intrinsic motivation in preclinical years, a decline during intermediate clinical training, and partial recovery in the final year (Figure 2). Binary logistic regression was significant, F(7, 1208) = 14.6, *p* < 0.001, explaining 7.8% of variance (R^2^ = 0.078). Program type was the strongest predictor (estimate = 2.369, OR = 10.68, *p* < 0.001), with students in the novel curriculum exhibiting higher motivation than those in the original program. Year of study negatively predicted motivation (estimate = −0.440, OR = 0.644, *p* < 0.001), suggesting reduced motivation in later years. Sex was also significant (estimate = −0.324, OR = 0.723, *p* = 0.050), with women showing higher motivation than men. Stress (*p* = 0.258), mental health diagnosis (*p* = 0.304), and age group (*p* = 0.769) were nonsignificant (all *p* > 0.05). Multicollinearity was acceptable (all VIFs < 5.3).

### 3.3. Academic Performance

The regression model was significant, F(7, 1208) = 14.6, *p* < 0.001, accounting for 7.8% of variance (R^2^ = 0.078). Program type was the strongest predictor (β = −0.612, *p* < 0.001), favoring the novel curriculum. Women outperformed men (β = −0.333, *p* < 0.001), while higher academic years were linked to lower performance (β = −0.286, *p* < 0.001). Greater motivation (β = 0.096, *p* < 0.001) and lower stress (β = −0.079, *p* = 0.008) predicted better performance, whereas mental health diagnosis was associated with reduced performance (β = −0.254, *p* < 0.001). Age was non-significant (*p* = 0.427).

### 3.4. Factors Related to Well-Being: Sex, Mental Health, and Stress

#### 3.4.1. Sex Differences

Chi-square analysis showed a significant association between sex and mental health status, χ^2^(1) = 22.1, *p* < 0.001, with women having higher odds of no diagnosis (OR = 0.563, 95% CI [0.442, 0.716]). Mann–Whitney U tests revealed sex differences in stress and performance: women reported higher stress (M = 7.04) than men (M = 6.13; U = 147,366, *p* < 0.001, rank-biserial r = 0.257) but better performance (M = 8.76 vs. M = 8.58; U = 143,646, *p* < 0.001, rank-biserial r = 0.155).

#### 3.4.2. Mental Health and Its Impact

**Figure 3:**
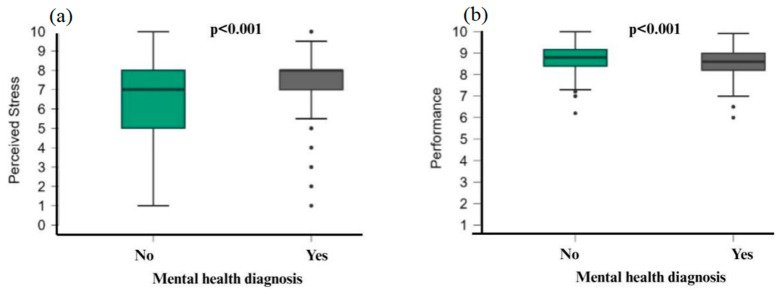
Mann–Whitney U test. (**a**) Perceived stress in students with mental health diagnoses, (**b**) performance of students with mental health diagnoses.

Students with a mental health diagnosis reported higher stress (M = 7.45, SD = 1.71) than those without (M = 6.27, SD = 2.05; U = 134,149, *p* < 0.001, rank-biserial r = 0.348; mean difference = −1.00, moderate effect). They also showed lower performance (M = 8.60 vs. M = 8.75; U not reported, *p* < 0.001, rank-biserial r = 0.146; small effect; Figure 3).

#### 3.4.3. Stress Across Academic Years

Kruskal–Wallis testing indicated significant differences in stress by year, χ^2^(4) = 34.064, *p* < 0.001. Dunn’s post hoc tests (Bonferroni-corrected) showed fourth-year students had higher stress than first- and final-year students (*p* < 0.001). Final-year students reported lower stress than third-year students (*p* = 0.053), suggesting a peak in year 4 and lowest point in the final year (Figure 2a).

### 3.5. Network Analysis of Academic Motivation

#### 3.5.1. Descriptive Statistics and Network Properties

Means and standard deviations for motivational factors are presented in Table 2. Intrinsic motivation to know had the highest mean (M = 17.44, SD = 3.32), while year of study had the lowest (M = 2.05, SD = 1.45). Amotivation showed the greatest variability (M = 7.12, SD = 3.73), and academic performance the least (M = 8.71, SD = 0.65). No redundant nodes (r > 0.50) were detected, indicating distinct variable relationships. All predictability values were significant (*p* < 0.001).

#### 3.5.2. Local Network Properties

**Figure 4:**
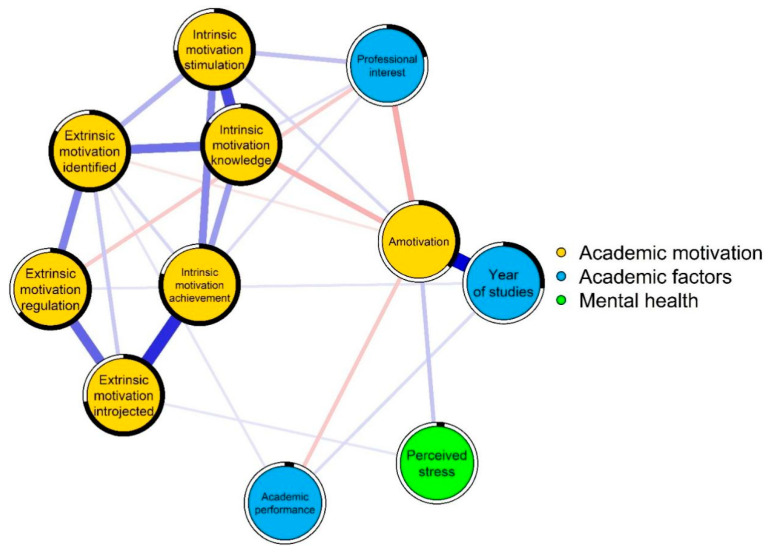
Network structure of academic motivation, academic factors, and mental health in medical students.

Intrinsic motivation for achievement was most central (strength index = 1.11), followed by intrinsic motivation for stimulation (1.07), extrinsic motivation identified (0.98), and extrinsic motivation introjected (0.98). Predictability was highest for intrinsic motivation to know (84.5%), extrinsic motivation identified (83.4%), and intrinsic motivation for achievement (79.4%) (Figure 4).

#### 3.5.3. Global Network Properties

The network included 11 nodes with density = 0.060, comprising 20 positive and 5 negative edges. Clustering (C△ = 0.47 vs. random = 0.41) and average path length (APL = 1.56 vs. random = 1.70) indicated efficient connectivity. The small-world index (1.25) confirmed balanced local clustering and global efficiency.

#### 3.5.4. Network Associations

**Figure 5:**
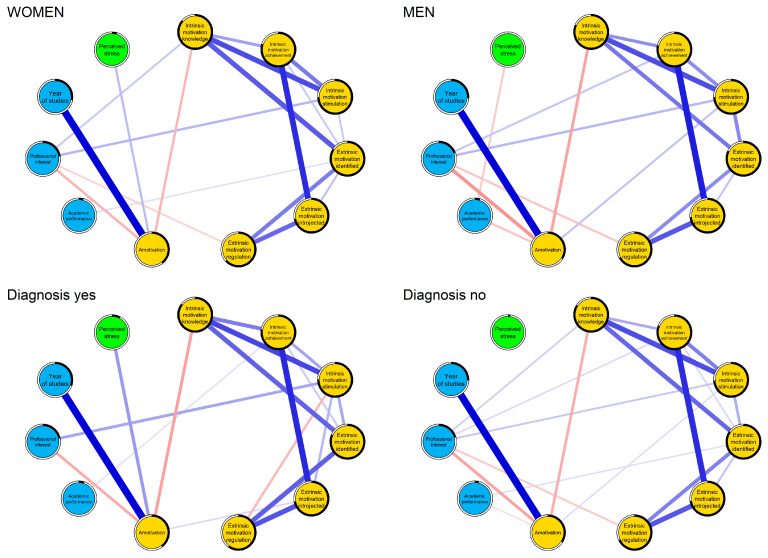
Comparison of network structures according to sex and mental disorder diagnosis. Yellow nodes correspond to academic motivation, light blue nodes to academic factors, and green nodes to mental health.

Figure 4 and Figure 5 depict the network of academic motivation, performance, and mental health. Strongest associations included amotivation with year of study (r = 0.56), intrinsic motivation for achievement with extrinsic motivation introjected (r = 0.46), intrinsic motivation to know with stimulation (r = 0.40), extrinsic regulation with introjected (r = 0.34), and intrinsic to know with identified (r = 0.32).

#### 3.5.5. Comparative Analysis of Network Structures

Networks were stratified by sex (women: n = 719; men: n = 400) and mental health diagnosis (diagnosed: n = 441; not: n = 678). High correlations existed between structures for sex (r = 0.93) and diagnosis (r = 0.91). No significant differences emerged in structure invariance (sex: M = 0.129, *p* = 0.452; diagnosis: M = 0.147, *p* = 0.193) or global strength (sex: S = 0.204, *p* = 0.769; diagnosis: S = 0.387, *p* = 0.466; (Supplementary Materials). Centrality stability (Supplementary Materials) supported connection reliability. Motivational structures were invariant across groups (Figure 5).

### 3.6. Qualitative Findings on Participation and Engagement

**Figure 6:**
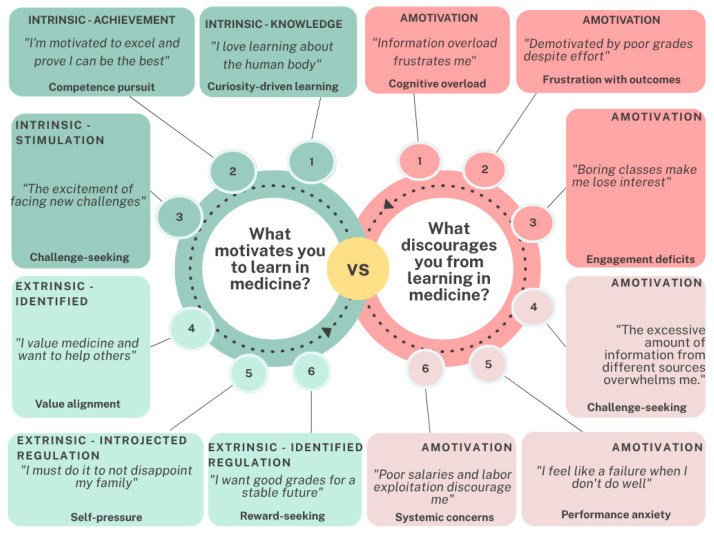
Student Motivation in Medical Education: Intrinsic and Extrinsic Factors.

Thematic analysis of open-ended responses highlighted affective motivations rooted in passion for patient-centered care, which provided purpose and meaning. However, barriers such as frustration from unachieved academic goals, excessive workloads, and suboptimal learning environments frequently eroded self-esteem and fostered demotivation (Figure 6).

The binary logistic regression model was statistically significant, F(7, 1208) = 14.6, *p* < 0.001, explaining 7.8% of the variance in motivation (R^2^ = 0.078). Program type was the strongest predictor (Estimate = 2.369, OR = 10.68, *p* < 0.001), with students in the novel program showing significantly higher motivation than those in the original program. Year of study negatively predicted motivation (Estimate = −0.440, OR = 0.644, *p* < 0.001), indicating lower motivation in higher academic years. Sex was a significant predictor (Estimate = −0.324, OR = 0.723, *p* = 0.050), with women showing higher motivation than men. Stress levels (*p* = 0.258), mental health issues (*p* = 0.304), and age group (*p* = 0.769) were not significant predictors of motivation. Stress, mental health, and age were non-significant (*p* > 0.05). Multicollinearity was acceptable (VIFs < 5.3).

The psychological network showed adequate accuracy in estimating edge weights and robust stability of expected influence centrality (CS coefficient = 0.52 for correlation stability, exceeding the recommended threshold of 0.5). Modeling the network of partial correlations between SDT needs, FPAE pillars, and performance indicators revealed robust centrality metrics, with competence emerging as the most central node and bridging symptoms, such as affective engagement, linking autonomy and social engagement. These findings extend the regression results by identifying intervention targets not considered by linear models, thus improving interpretive depth and complementing the project’s qualitative information (see Supplementary Material S1 for network graphs and stability metrics).

#### Qualitative Analysis of Motivational Factors

**Figure 7:**
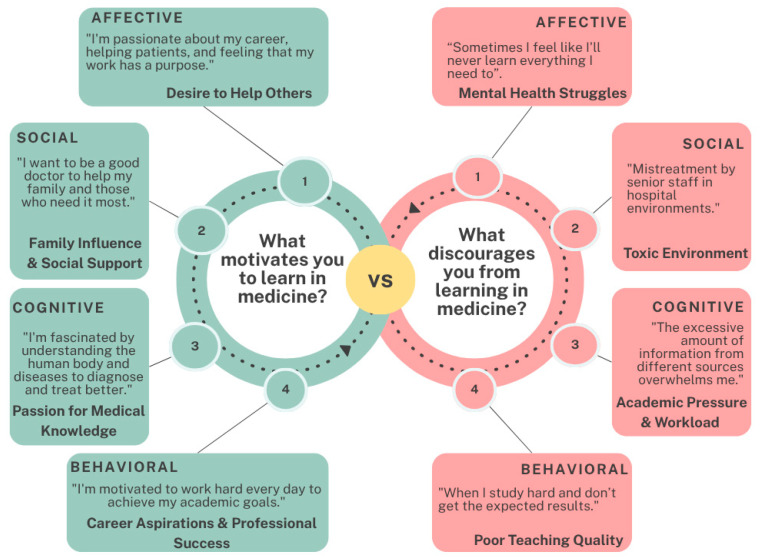
Student Engagement in Medical Education: Motivations and Barriers.

**Figure 8:**
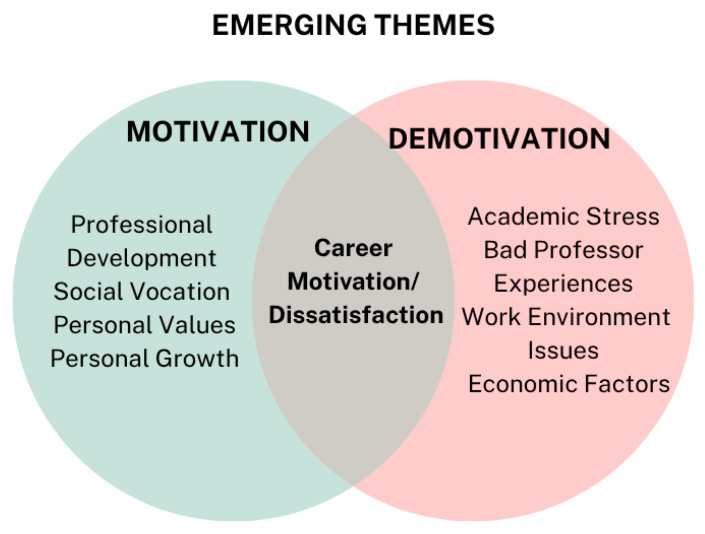
Student Academic Motivation: Emerging Themes.

Thematic analysis of open-ended responses identified key factors influencing student motivation in medical education. Intrinsic motivators, particularly a passion for understanding human physiology and its complexities, emerged as primary drivers. Additionally, students valued medicine as a means to serve others, honor family support, and secure a stable future, with these elements collectively representing the dominant motivational factors (see Figure 6, Figure 7 and Figure 8).

Regarding participation and engagement in medical education, affective motivations centered on a passion for patient-centered work that imbues professional activities with purpose and meaning. However, students frequently reported barriers, including frustration and disappointment stemming from an inability to achieve desired academic outcomes due to excessive workloads or perceptions of suboptimal learning environments. These challenges often undermined students’ self-esteem, contributing to demotivation (see Figure 7).

Derived from responses to the open-ended questions, the diagram categorizes themes into two overlapping circles, illustrating dissimilar and shared elements that influence engagement (Figure 8). These findings suggest that intrinsic motivators are critical in fostering sustained engagement, offering insights for designing interventions that connect with participants’ personal and professional aspirations.

## 4. Discussion

The present study examined the evolution of motivation among medical students across training stages, assessing the influence of curricular innovation, gender, and mental health on academic engagement, using SDT and the FPAE as theoretical frameworks (Bowden et al., 2021; Deci & Ryan, 2000).

Findings revealed substantial motivational variability: higher levels in preclinical years, a marked decline during clinical training (years 3–4), and partial recovery in the final year. This pattern aligns with previous evidence of motivational erosion during the transition to demanding clinical settings (Kusurkar et al., 2013) and is consistent with SDT, which posits that autonomy, competence, and relatedness are particularly threatened during intensive clinical rotations. The steepest decline, coinciding with peak stress in year 4, reflects the shift from structured preclinical learning to high-stakes clinical courses and evaluative pressure. Recovery in the final year is attributable to restored autonomy (e.g., clinical decision-making), enhanced competence through immersive practice, and deeper patient-related relatedness—all of which rekindle intrinsic motivation (Ommering et al., 2021; Sarkis et al., 2020; Spitzer et al., 2025).

Qualitative data highlighted altruism and intellectual curiosity as dominant motivators, especially when students perceived meaningful clinical impact. Unlike (Matos Sousa et al., 2025), who found no significant differences by gender or year of study, the present study identified significant variation by academic year and gender, though age showed no association.

Curricular design emerged as a powerful determinant of motivation. Students in the reformed curriculum—featuring elective courses and student-centered active methodologies (high- and low-fidelity simulation, gamification, flipped classroom, and case-based learning)—exhibited significantly higher motivation than those in the legacy program (*p* < 0.001). This finding underscores the critical role of curricular structure in fostering engagement (Storz et al., 2022) and is amplified by immersive technologies: augmented reality enhances empathy and practical competence (Chavez-Maisterra et al., 2024), virtual reality improves engagement and conceptual mastery in complex subjects (Toral-Murillo et al., 2024), and virtual laboratories boost motivation and critical thinking while overcoming logistical barriers (Zamora-González et al., 2024).

Surprisingly, no significant association was found between self-reported mental health issues and motivation, possibly due to robust institutional support services. However, peak stress in year 4—coinciding with the motivational nadir—emphasizes the need for targeted well-being initiatives to prevent burnout. Unexplored variables such as religiosity, family influence, and the emotional impact of being a non-local student may also modulate motivation.

Network analysis identified intrinsic motivation for accomplishment and knowledge as the most central nodes, confirming their pivotal role in engagement (Kusurkar et al., 2013). Amotivation was strongly linked to later academic years, reflecting cumulative disillusionment exacerbated by toxic competition and excessive workload, a perception corroborated by qualitative accounts. The network’s structural stability across gender and mental health status suggests universal motivational mechanisms, though their expression is modulated by contextual stressors. Mistreatment and poor teaching emerged as major barriers, consistent with prior Mexican studies highlighting distrust in reporting mechanisms (Olivares Olivares et al., 2021).

These findings carry important implications for medical education. Curricula that incorporate elective opportunities, immersive technologies, and student-centered active learning can mitigate the typical motivational decline during clinical training. Institutions should implement stage-specific interventions—autonomy-promoting electives in preclinical years and structured mentoring during clinical phases—alongside transparent reporting systems and cultural changes that ensure equity and psychological safety (Fallatah et al., 2018; Moreno-Tetlacuilo et al., 2016).

## 5. Study Limitations

This study has several limitations. Firstly, the models showed low explanatory power, omitting crucial predictors like socioeconomic status, sleep quality, mentorship quality, religiosity, or ideological beliefs, which interact complexly with motivation and engagement. Future research should incorporate these.

Secondly, the single-institution design at a private Mexican medical school restricts generalizability to public institutions, international settings, or programs with different admission criteria, student profiles, or curricula. This also introduces potential bias from differential exposure to the legacy versus reformed curriculum.

Thirdly, mental health data relied solely on self-report, lacking clinical verification, potentially leading to inaccurate reporting. Fourthly, the qualitative component was limited to open-ended survey responses, missing the richness of in-depth interviews or focus groups. Finally, the cross-sectional design prevents causal inferences and assessment of long-term effects of curricular innovations or the true developmental trajectory of motivation. To address these, future studies should employ longitudinal designs, probabilistic multisite sampling, clinically validated mental health measures, and mixed-methods approaches with richer qualitative triangulation. These efforts will strengthen causal claims and enhance the external validity of findings in medical education research.

## 6. Conclusions

Medical student motivation is dynamic, high in the preclinical years, declining sharply during the demanding clinical years (particularly the fourth year, which coincides with peak stress), and partially recovering in the final year. This decline could be largely due to the clinical transition, which often compromises students’ autonomy, competence, and interpersonal skills, increasing the risk of burnout. The curriculum appears to influence motivation; the new curriculum that included elective courses, active learning, and immersive technologies significantly boosted motivation compared to the former program. Throughout their training, altruism and intellectual curiosity serve as powerful intrinsic motivators, especially when students observe a tangible clinical impact. Ultimately, despite the existence of largely universal motivational mechanisms, contextual stressors such as curriculum, toxic competition, excessive workload, mistreatment, and poor teaching decisively influence student outcomes.

## Figures and Tables

**Table 1:** Coefficients for Binary Logistic Regression Predicting Motivation.

Predictor	Estimate (Log Odds)	SE	Z	*p*	OR	95% CI
Intercept	−1.163	0.300	−3.876	<0.001	-	-
Stress levels	−0.046	0.041	−1.131	0.258	0.955	[0.881, 1.036]
Mental health (1 vs. 0)	0.176	0.171	1.027	0.304	1.192	[0.852, 1.667]
Program (1 vs. 2)	2.369	0.350	6.77	<0.001	10.68	[5.37, 21.24]
Year of study	−0.440	0.113	−3.885	<0.001	0.644	[0.516, 0.803]
Sex (0 vs. 1)	−0.324	0.165	−1.961	0.050	0.723	[0.523, 1.000]
Age group (2 vs. 1)	−0.054	0.185	−0.293	0.769	0.947	[0.659, 1.361]

**Table 2:** Descriptive Statistics and Network Properties of Academic Motivation Factors.

Node	M	SD	Expected Influence	Predictability
Intrinsic motivation knowledge	17.44	3.32	0.83	84.5%
Intrinsic motivation achievement	16.32	3.31	1.11	79.4%
Intrinsic motivation stimulation	15.82	3.41	1.07	74.2%
Extrinsic motivation identified	17.43	3.29	0.98	83.4%
Extrinsic motivation introjected	16.28	3.64	0.98	72.2%
Extrinsic motivation regulation	16.34	3.41	0.56	64.3%
Amotivation	7.12	3.73	0.23	36.0%
Academic performance	8.71	0.65	0.02	3.5%
Professional interest	8.83	1.35	−0.01	21.4%
Year of studies	2.05	1.45	0.70	27.0%
Perceived stress	6.66	2.06	0.18	2.9%

M = Mean, SD = Standard Deviation, EI = Expected Influence (or centrality index), P = Predictability, all predictability values significant at *p* < 0.001.

## Data Availability

The database will be available for readers upon request.

## Supplementary Materials

The following supporting information can be downloaded at: https://www.mdpi.com/article/10.3390/bs16010097/s1. Figure S1. Nonparametric bootstrapping confidence intervals of estimated edges for the network structure. Figure S2. Stability of the IE centrality index.
