# Supplementary material for: Analysis of Medical Students’ Motivation: Insights into the Development of Future Health Professionals
Source: Behav Sci (Basel). 2026 Jan 12;16(1):97. doi: 10.3390/bs16010097 (PMC12838171; doi:10.3390/bs16010097)
Supplement: Supplementary file 1 [file behavsci-16-00097-s001.zip › behavsci-3746606-supplementary.pdf]

Supplementary Material S1

Comparative Analysis of Network Structures

No significant differences were identified in structural invariance (M=0.147, p=0.193) or overall strength (SI=0.387, p=0.466) (Figure 5).The stability of the centrality index (Figure 6) supports the reliability of the connections identified in the motivational network. Motivational structures were consistent across groups (sex and mental health diagnoses), indicating no significant differences in network configurations.

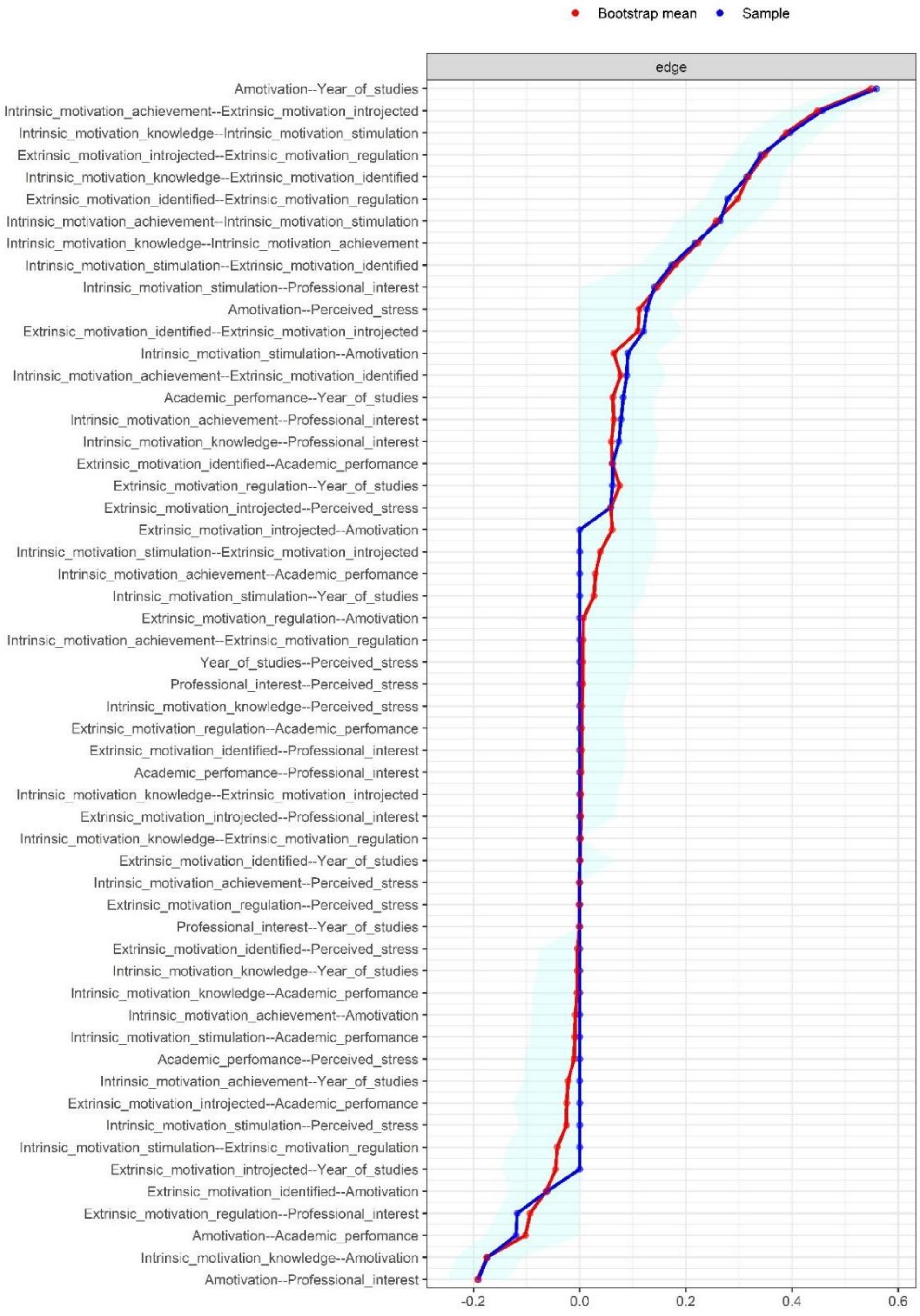


*Figure S1. Nonparametric bootstrapping confidence intervals of estimated edges for the network structure.*

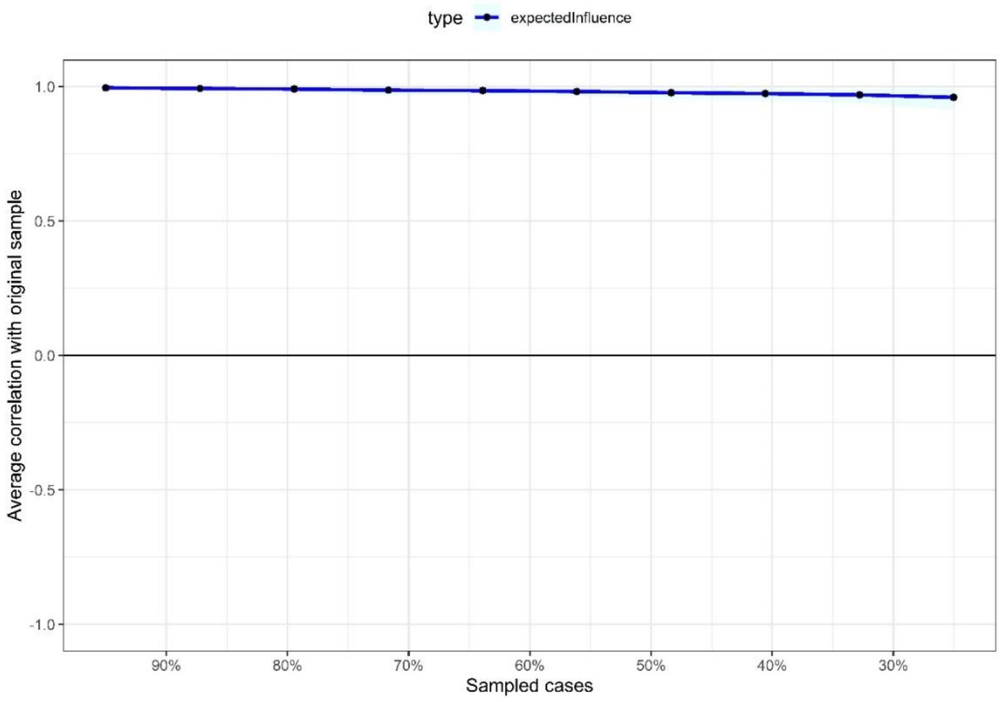


*Figure S2. Stability of the IE centrality index.*
